# Defect-Free Single-Layer Graphene by 10 s Microwave Solid Exfoliation and Its Application for Catalytic Water Splitting

**DOI:** 10.1021/acsami.1c03906

**Published:** 2021-06-10

**Authors:** Mustafa K. Bayazit, Lunqiao Xiong, Chaoran Jiang, Savio J. A. Moniz, Edward White, Milo S. P. Shaffer, Junwang Tang

**Affiliations:** † Department of Chemical Engineering, University College London, Torrington Place, London WC1E 7JE, U.K.; ‡ Department of Chemistry, Imperial College London, London SW7 2AZ, U.K.

**Keywords:** defect-free single-layer graphene, fast production, special mode microwave-intensified process, conductivity, oxygen evolution reaction, water splitting

## Abstract

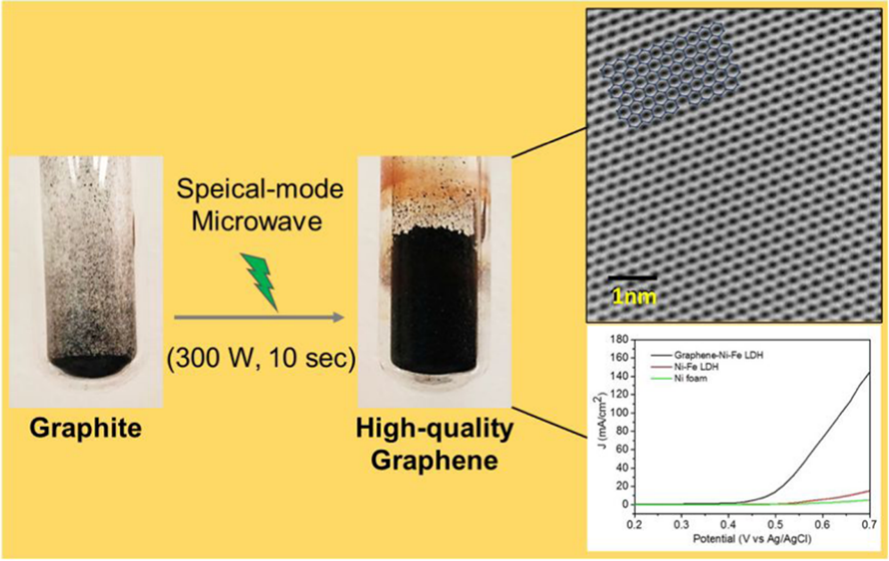

Mass production of defect-free single-layer graphene flakes (SLGFs) by a cost-effective approach is still very challenging. Here, we report such single-layer graphene flakes (SLGFs) (>90%) prepared by a nondestructive, energy-efficient, and easy up-scalable physical approach. These high-quality graphene flakes are attributed to a novel 10 s microwave-modulated solid-state approach, which not only fast exfoliates graphite in air but also self-heals the surface of graphite to remove the impurities. The fabricated high-quality graphene films (∼200 nm) exhibit a sheet resistance of ∼280 Ω/sq without any chemical or physical post-treatment. Furthermore, graphene-incorporated Ni–Fe electrodes represent a remarkable ∼140 mA/cm^2^ current for the catalytic water oxidation reaction compared with the pristine Ni–Fe electrode (∼10 mA/cm^2^) and a 120 mV cathodic shift in onset potential under identical experimental conditions, together with a faradic efficiency of >90% for an ideal ratio of H_2_ and O_2_ production from water. All these excellent performances are attributed to extremely high conductivity of the defect-free graphene flakes.

## 1 Introduction

The graphene-based technologies heavily rely on the availability of mass production of high-quality and low-cost single-layer material in a liquid-processable form,^1,2^ which still is a challenge.^3^ So far, physical approaches have been favored to produce relatively high-quality graphene flakes, the high cost and low product yields could be major obstacles limiting its scale-up.^4^ For example, liquid-phase exfoliation of graphite has widely been used to produce graphene flakes; however, it either produces multilayered products or the yield of the produced mono/bilayer graphene is not satisfactory due to the strong π–π interactions between the highly ordered graphitic layers.^5−10^ Increasing the ultrasonication time or intensity or applying a high shear force could improve the graphene production yield by weakening layer–layer interactions. However, these processes tend to create defects on the graphene surface, which significantly disrupt the electronic properties of the material and increases the manufacturing costs.^5,9−13^ In addition to the liquid-phase exfoliations, a gas-driven exfoliation of graphite was also shown to be effective in preparing monolayer flakes with a yield of 62%.^14^ Furthermore, conventional thermal expansion of intercalated graphite compounds at high temperatures (*e*.*g*., 800 °C) under inert conditions was shown to be useful to prepare bi- and trilayer graphene solutions as the major products after sonication.^15^ However, short-term stability of these intercalated graphite materials required immediate processing just after preparation besides high-energy input required. Thus, chemical approaches are always believed to be a low-cost technology but the resultant graphene products usually suffer from unavoidable surface defects and low yields.^16−18^ A benchmark work using advanced chemical reduction of highly defective graphene oxide successfully improved the quality of graphene flakes with still ∼7 atom % total oxygen (in-plane oxygen and noncovalently bonded adsorbed oxygen) and a few milligrams of yield.^19^ Similar phenomena were reported.^20,21^

Microwave irradiation (MI), in particular, operated in special mode, provides rapid, noncontact, volumetric, and selective heating by directly interacting with a material with high dielectric loss. The commercially available low-cost graphite is just such a candidate that exhibits a large dielectric loss or is highly conductive.^22^ Furthermore, microwave radiation is known to be an effective nondestructive technique to remove oxidative surface moieties on the graphitic framework.^19^ A solution phase high-yielding exfoliation (yield of ∼93%) of graphite was achieved in an expensive solvent, a highly fluorinated oligomeric ionic liquid,^23^ or similarly by other methods, *e*.*g*., electrochemical exfoliation^24^ or nondispersion exfoliation.^25^ The mechanism of the microwave process was attributed to the high-temperature decomposition of expensive solvent into highly toxic hydrogen fluoride, which concurrently intercalated the graphite layers. However, a nondestructive solvent-free physical approach to manufacture high-quality single-layer graphene flakes (SLGFs) is highly sought for both academically and industrially as it does not have an impact on the environment and more importantly promises a high-quality product. Herein, a microwave irradiation (MI) is strongly coupled with graphite to efficiently weaken the strong π–π interactions of graphitic layers in solid state in air within a very short time of ∼10 s. The MI-assisted nondestructive and irreversible solid-state expansion/exfoliation of graphite yields high-quality graphene with excellent physical and chemical properties. More importantly, such high-quality graphene flakes can improve the water splitting efficiency by a factor of 10 and also shows a cathodic shift of the onset potential by 120 mV. The fundamental studies prove the special strength of the special mode MI-promoted approach and the advantage of the produced graphene flakes.

## 2 Results

**Figure 1:**
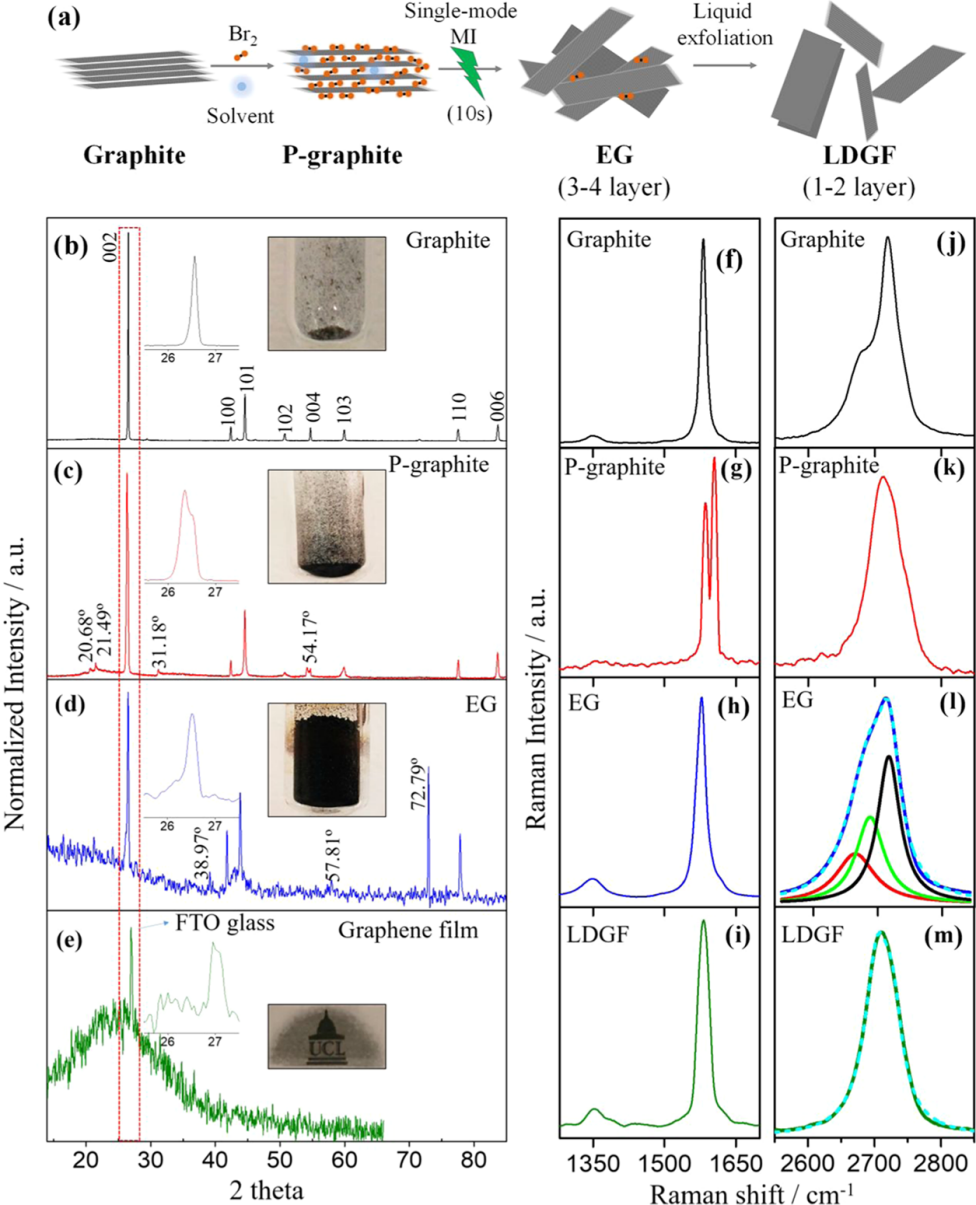
Special mode microwave-controlled nondestructive and irreversible solid-state exfoliation of graphite into graphene and the corresponding characterization by X-ray diffraction (XRD) and Raman spectroscopy. (a) This scheme illustrates three steps: the room-temperature treatment of the graphite in a Br_2_/CHCl_3_ solution to produce the P-graphite, then exfoliation of the P-graphite to yield the EGs by about 10 s MI, and finally the LDGFs. (b–e) X-ray diffraction patterns of the graphite, P-graphite, EG, and the graphene film (∼200 nm) on fluorine-doped tin oxide (FTO) glass prepared using the LDGFs in dimethylformamide (DMF). The insets are the expanded strongest peak regions of the corresponding graphs. Note that the change in the peak position and shape of the 002 reflection together with the appearance of new peaks labeled in corresponding XRD patterns indicate *d*-spacing alteration after Br_2_ pretreatment and the MI exfoliation. The inset photographs show the graphite (40 mg), P-graphite, EGs, and graphene film. (f–i) D- and G-band regions and (j–m) 2D-band region Raman spectra of the graphite, P-graphite, EG, and the LDGFs in DMF, respectively. The 2D-band region Raman spectra of the EG and LDGFs were fitted by a Lorentzian function. Dashes lines shown on the original spectrum (solid line) refer to fit line. Analyses were carried out on a Si-wafer surface using a Raman laser excitation of 514 nm. The D- and G-bands are observed in the region between 1200 and 1700 cm^–1^.

Figure 1a illustrates three key steps in the high-quality graphene synthesis: the room-temperature pretreatment of the graphite in a bromine/chloroform (Br_2_/CHCl_3_) solution to produce the P-graphite, then exfoliation of the preactivated graphite (P-graphite) to yield the EGs by 10 s MI, and finally the liquid-dispersed graphene flakes (LDGFs). All parameters affecting the final product quality were thoroughly investigated and are summarized in the Supporting information (SI). First, different solvents and treatment periods used in the pretreatment process were investigated and are presented in SI Figure S1. We found that the pretreatment in Br_2_/CHCl_3_ solution for 2 h produced stable P-graphite. It is also worth noting that this P-graphite can be stored in a sealed glass container in air and can still yield high-quality EGs by MI even after a year of storage. In parallel, a control experiment was carried out, where the as-received graphite was irradiated under identical experimental conditions using MI, which showed no volume expansion although the temperature of the as-received graphite was increased to ca. 360 °C. These findings clearly confirm the function of Br_2_ in the special mode microwave-controlled solid-state (MCSS) approach. In a separate control experiment, the P-graphite was subjected to a shock heating at 400 °C in a tube furnace under either nitrogen or airflow. Under both conditions, the volume of the P-graphite initially expanded just after placing it in the hot zone; however, the starting small volume was retained after cooling down to room temperature, suggesting that stable thermal expansion of the P-graphite cannot be achieved even till 400 °C using conventional heating and an irreversible exfoliation process can only be achieved by MI. Therefore, these control experiments clearly confirm the synergy between MI and Br_2_ treatments for efficient and stable exfoliation of graphite particles.

The most intense peak at *ca*. 26.50° in the XRD pattern of the P-graphite (Figure 1c and inset graph) splits and shifts to lower 2θ values compared to the 002 plane of the graphite (Figure 1b and inset graph). The XRD pattern of the P-graphite also shows four new peaks at *ca*. 20.68, 21.49, 31.18, and 54.17°. Specifically, the new peak at *ca*. 20.68° corresponds to the *d*-spacing of 4.27 Å, which is in very good agreement with the reported sandwich thickness (4.24 Å) for graphite-Br_2_ compounds.^26,27^ The XRD pattern of the EG exhibits three new peaks positioned at 38.97° (weak), 57.81° (weak), and 72.79° (strong) (Figure 1d). The strongest peak shifts back and is identical to that of the graphite. Furthermore, the four new peaks in the P-graphite disappear, indicating that Br_2_ molecules are likely to have been completely removed by MI.

Interestingly, the 006 plane observed at *ca*. 84° for the graphite and P-graphite samples disappears and the strongest peak (002) also becomes relatively weak (please see the insets) in the XRD pattern of the EG, suggesting that the EG is probably composed of less than six graphitic layers in thickness, which is consistent with previous reports.^15^ The change in volume of graphite, P-graphite to EG, and the removal of Br_2_ molecules can be seen in the inset pictures (Figure 1b–d). The transparent graphene film produced using the LDGFs displays a very broad XRD peak at 2θ of *ca*. 26.00°, corresponding to an interlayer spacing of *ca*. 0.34 nm,^28^ together with a sharp peak from the FTO glass at 26.95° (Figure 1e and insets therein). The Raman spectrum of the P-graphite shows characteristic harmonic peaks of Br_2_ at *ca*. 240 cm^–1^ (ω_0_, strongest), 322 cm^–1^ (2ω_0_), 472 cm^–1^ (3ω_0_), and 706 cm^–1^ (4ω_0_) in comparison to that of the graphite, indicating that Br_2_ molecules were inserted into the graphite (SI Figure S4a).^29^ Upon careful inspection of the P-graphite Raman spectrum (Figure 1g), a slight blue shift and split G-band with relatively similar intensity at *ca*. 1585 cm^–1^ (*G*_1_) and 1604 cm^–1^ (*G*_2_) are observed, compared to the G-band of the graphite (*ca*. 1580 cm^–1^), which corresponds to the in-plane vibration of sp^2^-hybridized carbon atoms (Figure 1f).^30^ After the MI exfoliation, the G-band splitting disappears and the characteristic G-band of the graphite reappears in the Raman spectrum of the EG (Figure 1h), indicating removal of the intercalated Br_2_ molecules during the few seconds of MI irradiation, which is further evidenced by the disappearance of the characteristic Raman peaks of Br_2_ molecules (SI Figure S4a). The D-band at *ca*. 1350 cm^–1^ is widely used as a guide of surface defects and chemical functionalization, while the intensity ratio of D-band to G-band (*I*_D_/*I*_G_) is often used to indicate the concentration of defects within a graphitic material.^31^ The EG exhibits an *I*_D_/*I*_G_ ratio of 0.09, compared to 0.04 obtained for the graphite, indicating that the MI exfoliation step introduces relatively few additional defects. After mild sonication in DMF, the obtained LDGF exhibits an *I*_D_/*I*_G_ ratio of 0.07, almost identical to the *I*_D_/*I*_G_ ratio of EG and graphite, confirming the presence of defect-free graphene flakes (Figure 1i). The characteristic 2D-band of graphene is attributed to a two-phonon double resonance process, which broadens with increasing number of graphene layers.^32^ The 2D-band of the P-graphite (Figure 1k) displays a narrow (full width at half-maximum (FWHM) ∼ 59) but intense peak (*I*_2D_/*I*_G at 1585 cm^–1^_ = 0.62) at *ca*. 2715 cm^–1^ compared to the graphite (Figure 1j), which has an FWHM of ∼61 and an *I*_2D_/*I*_G_ ratio of ∼0.46 at *ca*. 2726 cm^–1^, indicative of a change in *d*-spacing after Br_2_ treatment.^32,33^ In contrast, no substantial change is observed in the *I*_2D_/*I*_G_ ratio (*ca*. 0.48) of the EGs (Figure 1l). However, there is a clear change in the 2D-band peak shape averaged from 48 EG flakes, which can be fitted by three Lorentzian peaks and be attributed to the production of three- to four-layer graphene flakes, which is in good agreement with the spectra of the reported four-layer graphene flakes.^34^ The 2D-band Raman spectrum of the LDGFs shows the characteristic Raman peak shape of bilayer graphene and can be fitted by two Lorentzian peaks (SI Figure S4b).^34^ These LDGFs exhibit an increased *I*_D_/*I*_G_ ratio of 0.35, comparable with the *I*_D_/*I*_G_ ratio of edge-functionalized small graphene flakes (*I*_D_/*I*_G_ ∼ 0.35), probably due to the sonication process.^35^ It is worth mentioning that these bilayer flakes formed by dispersion of three- to four-layer EG in DMF are likely to be formed by the restacking of SLGFs during the sample preparation for Raman analysis as high-resolution transmission electron microscopy (HRTEM) results clearly show the presence of SLGFs (see below) because the Raman spectra of many individual flakes of LDGFs clearly display the *I*_2D_/*I*_G_ ratio of ∼2 (Figure 1m), in good agreement with previously reported SLGFs.^15,32^

The 2D-band maxima of these LDGFs is shifted to *ca*. 2700 cm^–1^ and can be fitted with a single Lorentzian peak. However, they show a broad FWHM of ∼56, which may be attributed to rotationally reordered SLGFs,^34^ which strongly decouples the electronic states of adjacent graphene layers, sustaining a density of states similar to the individual SLGFs.^36^

**Figure 2:**
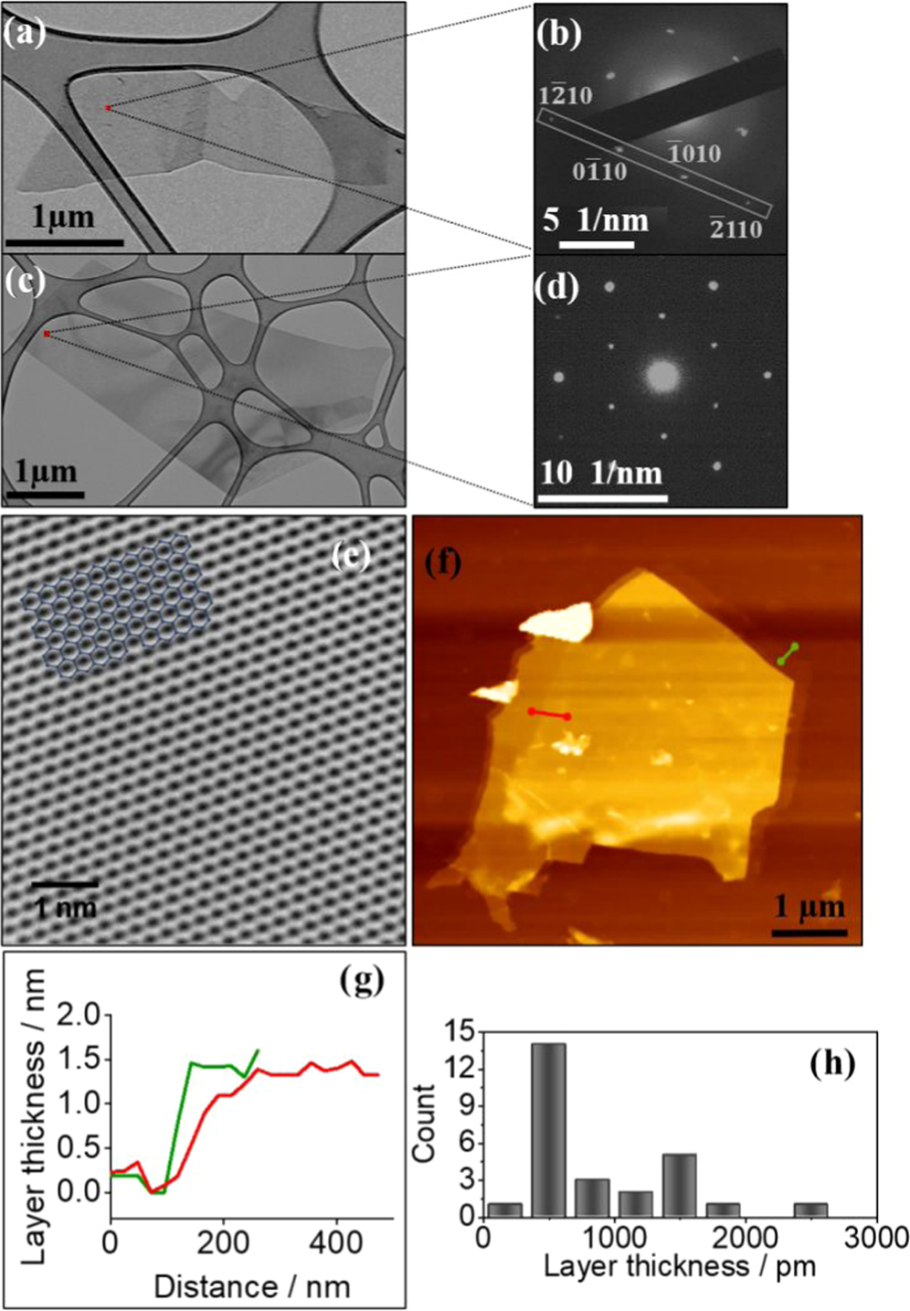
High-resolution transmission electron and atomic force microscopy (AFM) for single- and bilayer graphene classifications. (a) HRTEM image of an SLGF with a lateral size of ∼3 μm. (b) Selected-area electron diffraction (SAED) pattern taken from the position designated by the red square in a. Bright dots are labeled as (12̅10)–(01̅10)–(1̅010)–(2̅110) Miller indices. It can be observed that (01̅10)–(1̅010) diffractions are more intense (nearly double) compared to the (12̅10)–(2̅110) diffractions, indicative of SLGF. (c) HRTEM image of bilayer graphene flakes (BLGFs) with a lateral size of ∼5 μm. (d) SAED pattern taken from the position designated by the red square in (c). More intense (12̅10)–(2̅110) diffractions, compared to (01̅10)–(1̅010) diffractions, indicating double-layer graphene. (e) HRTEM image of graphene, which was performed on an aberration-corrected FEI Titan HRTEM operating at 80 kV. The image clearly shows a defect-free hexagonal lattice, providing further evidence that the MCSS approach is nondestructive, resulting in defect-free graphene flakes. The inset shows the location of hexagonal rings. (f) Typical AFM height image of the LDGFs. (g) Measured height of stacked flakes in (f), by step analysis, marked by straight lines (red and green) with oval-arrow heads. (h) Layer thickness distribution of LDGFs obtained by measuring the height of 27 randomly selected graphene sheets. The solution analyzed for optical characterization was used for TEM and AFM analyses.

Figure 2a visually confirms the presence of isolated SLGF with a lateral size of ∼3 μm. The selected-area electron diffraction (SAED) image shows the characteristic hexagonal pattern with diffraction peaks corresponding to the (12̅10)–(01̅10)–(1̅010)–(2̅110) Miller indices (Figure 2b), and the intensity of these diffractions is consistent with the SLGF previously reported.^10,23^

Larger bilayer graphene flakes (∼5 μm) are also observed in the analyzed dispersion (Figure 2c,d). HRTEM further confirms the presence of a high-quality, almost defect-free hexagonal lattice (Figure 2e), in line with the high-quality graphene reported but by more costly technologies.^19,37^ (SI Figures S5 and S6 for additional HRTEM images). AFM analysis shows graphene flakes with lateral dimensions of ∼0.5 to 5.0 μm (Figure 2f) and thicknesses of ≤1.5 nm (Figure 2g), comparable with the characteristic SLGF thickness reported in the literature.^10^ A detailed analysis of these flakes shows 0.5–1.5 nm of layer thickness distribution of LDGFs obtained by measuring the height of 27 randomly selected graphene sheets in the AFM image (Figure 2h and SI Figure S7).

**Figure 3:**
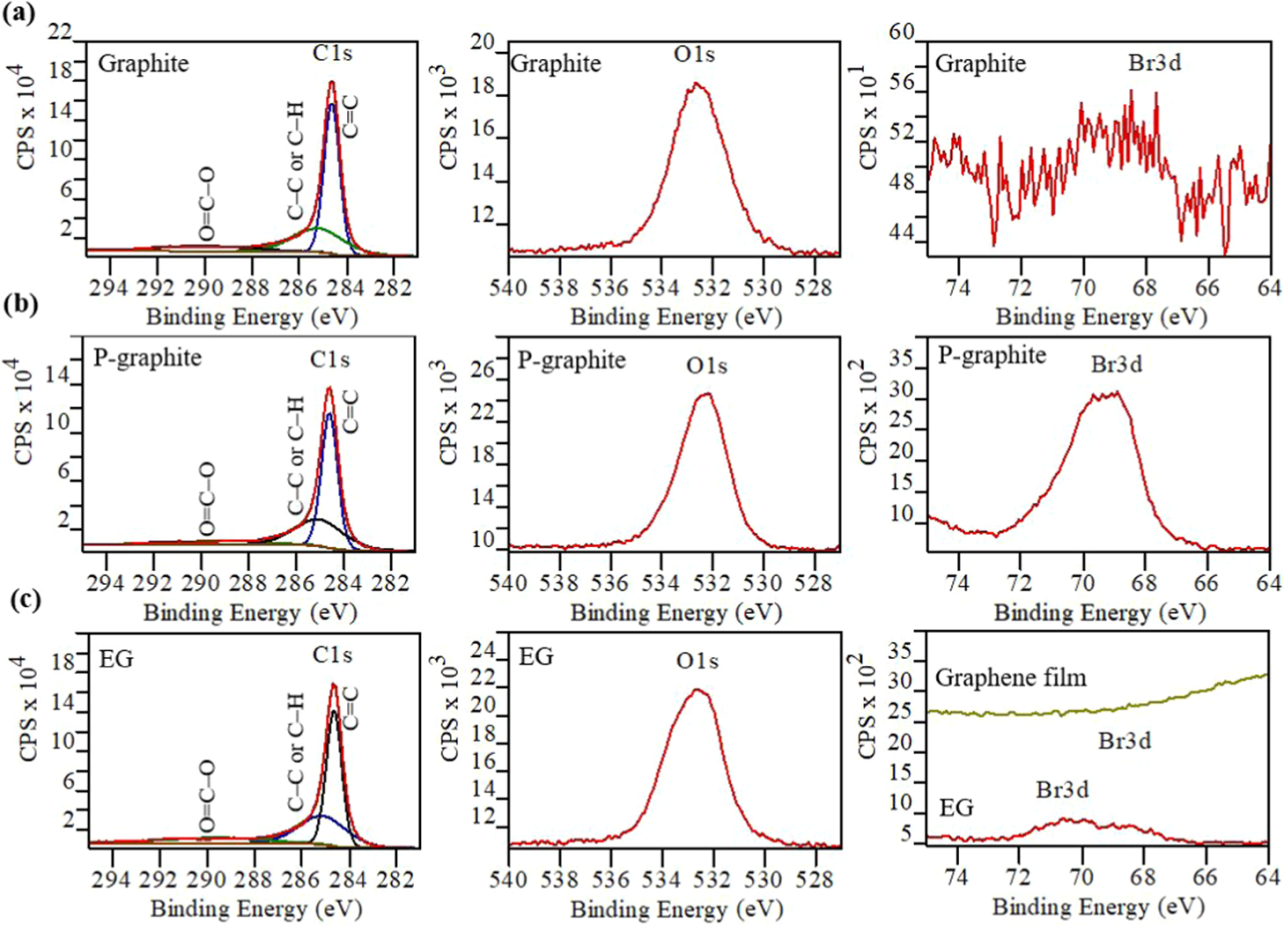
Monitoring the effect of the MCSS approach on the surface properties of the graphene flakes by X-ray photoelectron spectroscopy. (a–c) XPS C 1s, O 1s, and Br 3d spectra obtained for the (a) graphite, (b) P-graphite, and (c) EG and graphene film (upper line). Fitted lines in the C 1s XPS spectra are characterized as follows: sp^2^ C=C, sp^3^ C–H, and O=C–O. In all cases, the Shirley background was used. All spectra were calibrated by referring to the binding energy of C 1s = 284.5.0 eV. The binding energy range between ∼286.0 and 293.0 eV in the C 1s XPS spectra represents oxidized carbon components. XPS spectra clearly prove that this is a nondestructive MI solid-state exfoliation process.

Maintaining a pristine surface is regarded as a key factor dominating the graphene’s ideal properties for practical applications, *e*.*g*., in electronics, sensors, and energy storage. Thus, the complete removal of any surface impurities is highly desirable since they will potentially behave as impurity dopants, detrimental to the functional properties. Consistent with the Raman spectra and XRD, X-ray photoelectron spectroscopy (XPS) analysis further corroborates that both the pretreatment and the MI exfoliation processes are almost nondestructive, which preserves the sp^2^ lattice (Figure 3a–c). The P-graphite shows peaks related to C 1s, O 1s, and Br 3d at *ca*. 283.0–292.0, 530.0–535.0, and 66.0–73.0 eV, respectively (Figure 3b), compared to the main peaks of C 1s and O 1s in the graphite (Figure 3a). In addition, the graphite exhibits an undefined peak in the XPS region of Br 3d (at *ca*. 66.0–73.0 eV). After 10 s of microwave treatment, the EG exhibits an approximately 92% less intense Br 3d peak (0.29 atom % Br compared to 3.30 atom % Br in P-graphite) together with the other characteristic peaks of C 1s and O 1s (Figure 3c). Furthermore, the additional washing associated with the preparation of graphene film (see Figure 1e inset photograph) appears to remove all of the very small fraction of remaining residual Br_2_, indicating that it is weakly bound (Figure 3c). Overall, the MCSS approach not only exfoliates the P-graphite but also removes approximately 95% of the Br_2_ intercalate (estimated by XPS) in just 10 s, yielding almost contaminant- and defect-free EG without the need of a postpurification step, which is normally crucial when using other synthetic methods with different intercalates (*e*.*g*., metal salts,^19^ Brønsted acids,^13^ Lewis acids,^38^ etc.). Consistent with the literatures,^23,39^ the deconvoluted C 1s XPS spectra of the graphite exhibit three peaks at 289.0 (Cont. 18.1%), 285.2 (Cont. 26.9%), and 284.6 (Cont. 54.6%) eV, attributed to O=C–O, sp^3^ C–H, and sp^2^ C=C, respectively (Figur3^3^a).

Similar to this, the P-graphite and EG also show C 1s peaks with the same binding energies, suggesting that the chemical environment of C atoms in all materials is identical.^10^ Interestingly, on closer inspection of the O=C–O-related peak at *ca*. 289.0 eV (Cont. 18.1%) in the graphite, a decrease in the concentration of the O=C–O peak to 11.4% after the MI exfoliation is noticed. Compositional analysis by XPS further confirms half of the total amount of oxygen in the EG (∼95.3 C% and 4.7 O%), compared to approximately 91.8 C% and 8.2 O% in the graphite. Furthermore, the deconvoluted O 1s XPS spectra of the graphite, P-graphite, and EG are found at the same binding energies, all consisting of a major peak at *ca*. 532.4 eV.

Such 4.7% oxygen is believed to be adsorbed O_2_, which is present for both the original graphite and EG as commonly observed even after annealing at 1100 °C;^40^ therefore, the similarity between the signals before and after treatment suggests that no additional functionalization occurs as also proved by HRTEM analysis and XPS. These findings also suggest that MI more effectively removes oxygen-containing surface species (*e*.*g*., covalently attached oxygenated surface moieties) from the graphitic material surface than that reported recently.^19^ These results are also in good agreement with the Raman spectra, which show similar D-bands in both the EG and the graphite.

In summary, the prepared P-graphite was irradiated by the MI for about 10 s to obtain solid-state exfoliated and equally importantly Br_2_-free graphene flakes (EGs) as the residual Br_2_ was nearly completely removed by the fast microwave heating as indicated by the XPS. The solvent-free MCSS system is thus able to produce EGs (3–4 layer graphene flakes) with a yield of 100% (based on the amount of P-graphite loaded) in 10 s. In this respect, the efficiency of the MCSS is higher than those of the others with similar several-layer graphenes produced such as by electrochemical exfoliation (yield of ∼65%)^24^ or nondispersion exfoliation (yield of ∼82.5 wt %).^25^ Furthermore, the produced EGs are defect-free when compared to those produced by the abovementioned methods. After the final step of sonication, 2% production of SLGFs can be obtained in one run, compared with the previous method that produced ∼1 wt % of monolayer graphene.^10^ Although to some extent, this new method is not superior to a few monolayer production methods such as by energy-intensive gas-driven exfoliation (62%)^14^ or solution-state exfoliation by MI (∼93%),^23^ the obtained SLGFs herein are defect-free by a simple, energy-efficient, and green method that is much better than those prepared by the extremely strong exfoliation. On the other hand, a strong sonication of the as-received natural graphite or P-graphite without microwave exfoliation only produces a few layer graphene flakes with a low concentration of 5.1 ± 0.86 and 3.8 ± 0.4 μg/mL, respectively.

**Figure 4:**
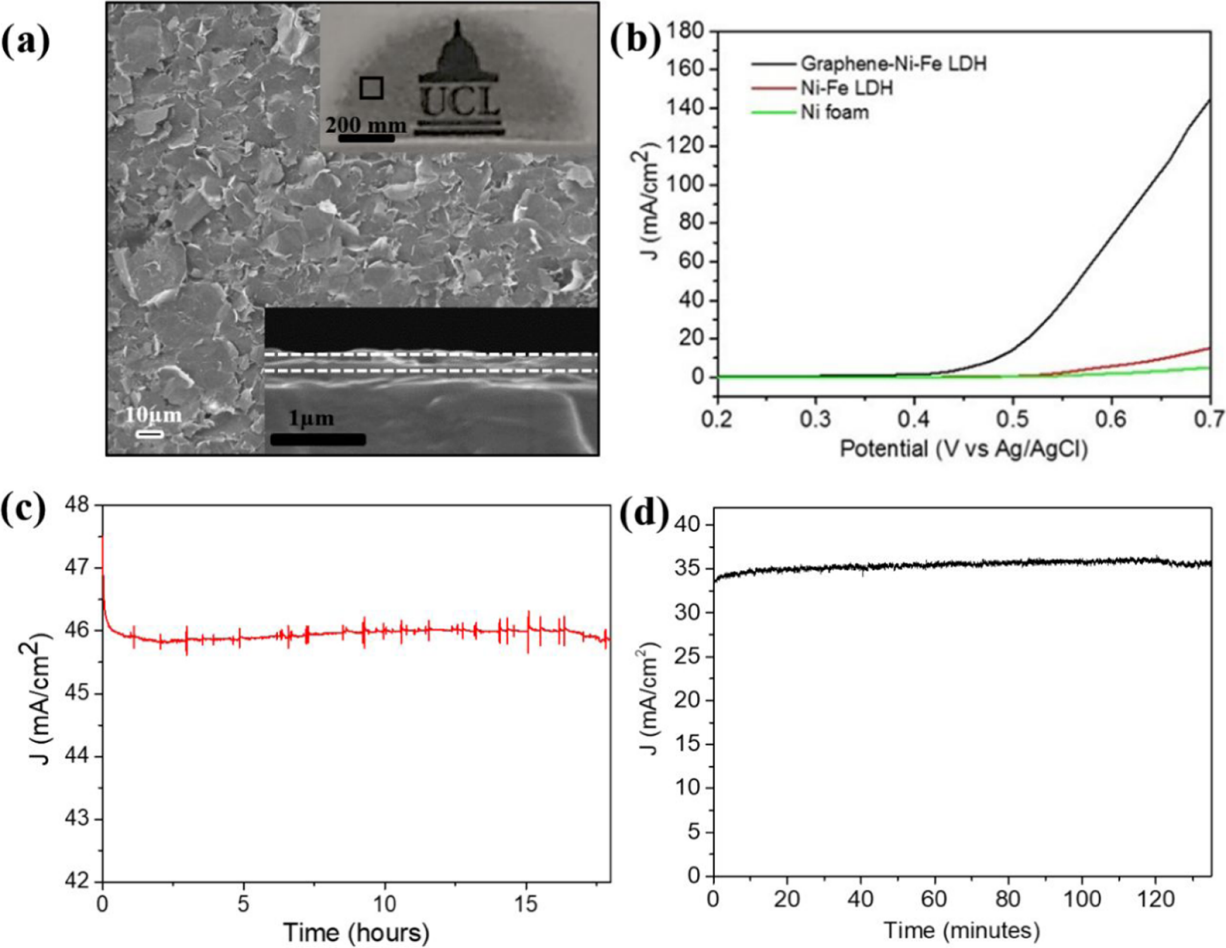
Physical and electrocatalytic properties of transferable graphene thin films. (a) scanning electron microscopy (SEM) image of the graphene thin film prepared *via* vacuum filtration on a PTFE membrane. Image was recorded on the PTFE membrane. The inset (top) shows the photograph of a graphene thin film prepared *via* vacuum filtration on a PTFE membrane and transferred onto a fluorine-doped tin oxide (FTO) glass substrate. The UCL logo beneath the glass slide coated with the graphene thin film (*ca*. 200 nm thickness) is clearly visible, indicating that the transferred film is at least semitransparent. The inset (below) shows the SEM cross-sectional image of the transferred graphene thin film deposited on the glass FTO substrate. White lines are used to show the thickness of the transferred film. (b) Current–voltage curves of Ni–Fe LDH and graphene–Ni–Fe layered double hydroxide (LDH) deposited on nickel foam together with pure nickel foam for the oxygen evolution reaction (OER) carried out in 1 M KOH (pH 14). (c) Electrochemical stability of graphene–Ni–Fe LDH on Ni foam for OER conducted at an applied potential of 0.58V *vs* Ag/AgCl for 18 h under constant stirring in 1 M KOH. The background noise is due to vigorous stirring and high sensitivity of the potentiostat. (d) Stable current density measured at an applied potential of 0.53 V *vs* Ag/AgCl for graphene–Ni–Fe LDH obtained during H_2_ and O_2_ evolution over a 2 h period with constant stirring.

The high-quality graphene with enhanced electrical properties has the strong potential to improve electron transport between the support and the catalyst, increasing catalytic activity.^41^ To evaluate the electrical properties of the high-quality graphene prepared here, we have studied the sheet resistance and the corresponding conductivity of the graphene films produced using varying volumes of LDGF dispersion (SI Figure S8a–c). Transferable graphene films were produced on PTFE membranes (SI Figure S8b) by vacuum filtration of 6, 3, and 1.5 mL stable dispersions of the LDGFs, washed with water, and dried at room temperature. The film was found to be composed of homogeneously oriented graphene sheets (Figure 4a). The film quality, which is always a key challenge in assessing its advanced practical application,^42^ was then evaluated. Prepared graphene films were analyzed by a four-probe conductivity meter without being subjected to any chemical or physical post-treatment (such as annealing) (Table 1). Measurements were taken from at least six different locations, which resulted in small sheet resistances of approximately 280 Ω/sq for the film (∼200 nm, ∼60% transmittance at 550 nm) prepared by filtering 6 mL of LDGFs compared to the reported sheet resistance (760 Ω/sq) of graphene films with a similar thickness of 200 nm^43^ produced by the nitronium ion-enabled method.

**Table 1:** Electrical Properties of the Fabricated Graphene Films^a^

	sheet Resistance (Ω/sq)			conductivity (S/m)		
solvent	6 mL	3 mL	1.5 mL	6 mL	3 mL	1.5 mL
DMF	2.80 × 10^2^	2.22 × 10^3^	1.15 × 10^5^	18 000	4504	174

a Sheet resistance and conductivity data for the graphene films prepared using 6, 3, and 1.5 mL stable dispersions of the LDGFs (10 μg/mL).

The conductivity of the film was significantly higher than that prepared by strong physical exfoliation and post-treatment (*e*.*g*., 5^10^ and 35 S/m^9^ before annealing; 5000^10^ and 1500 S/m^9^ after annealing). It is noted that there is air embodied in the film prepared here as no vacuuming was undertaken during film fabrication, which to some extent reduces the electrical conductivity, even though the measured conductivity is still comparable with the graphene films reported with a 50 times larger thickness.^5^ In addition, the prepared graphene films were easily transferrable onto various substrates such as glass (Figure 4a) and polystyrene (SI Figure S8c) *via* an ethanol-facilitated hot-stamping technique. The UV–vis spectra of the typical graphene film transferred to the FTO glass and uncoated FTO glass as a reference are shown in SI (Figure S8d).

The high-quality graphene was further tested in a typical oxygen evolution reaction (OER) in water splitting, which is the rate-determining step.^44^ Nickel iron layered double hydroxides (Ni–Fe LDH) and graphene–Ni–Fe LDH were deposited onto nickel foam substrates using a hydrothermal method (SI for details).^45^ Comparison of the OER properties of Ni–Fe LDH and graphene–Ni–Fe LDH reveals that the OER onset potential shifts cathodically by roughly 120 mV and the current density increases remarkably by more than an order of magnitude (at ∼0.7 V *vs* Ag/Ag/Cl) upon the addition of graphene as a highly conductive support, as shown in Figure 4b. Furthermore, the stability of the graphene–Ni–Fe LDH hybrid electrode was monitored at 0.58V *vs* Ag/AgCl and one can see that an extremely stable current of 46 mA/cm^2^ is represented without noticeable change over 18 h (Figure 4c). Such high current is believed due to the enhanced reaction kinetics by the graphene flakes produced by the special mode microwave-controlled nondestructive and irreversible solid-state exfoliation of graphite.

To verify that the high current is due to the electrical water splitting, the gaseous products were measured from the cell headspace while applying a constant external voltage of 0.53 V *vs* Ag/AgCl (Figure 4d). The electrode-exposed area was 0.5 cm × 0.5 cm, and 1253 μmol H_2_ and 626 μmol O_2_ were collected after 2 h of water electrolysis, corresponding to a faradic efficiency of ca. 91% with an ideal ratio of evolved H_2_ to O_2_ of 2:1. As the measurement was conducted in a small single cell reactor, where there was no separation of H_2_ and O_2_, gas mixing and the backreaction led to an underestimate of the actual gas evolution and loss of the faradic efficiency.

**Figure 5:**
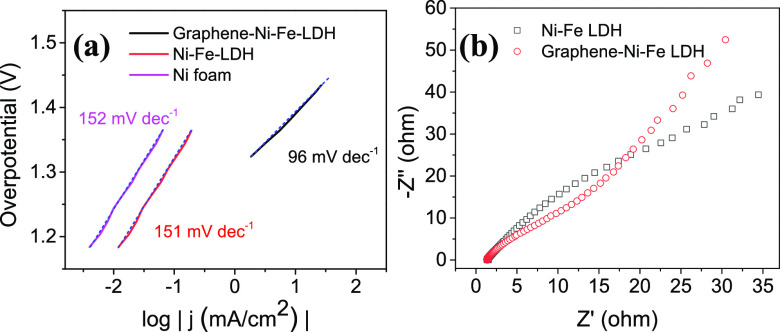
Reaction kinetics for oxygen evolution. (a) Tafel plots of Ni foam, Ni–Fe LDH, and graphene–Ni–Fe LDH. (b) The Nyquist plots of Ni–Fe LDH and graphene–Ni–Fe LDH.

In addition, the reaction kinetics for oxygen evolution was investigated by Tafel plots (Figure 5a). The Tafel slope value of graphene–Ni–Fe LDH is lower than that of Ni–Fe LDH, revealing its favorable reaction kinetics.^46^ The electrode kinetics of these catalysts in the OER process was also investigated by electrochemical impedance spectroscopy (EIS) measurements (Figure 5b). The impedance parameters by fitting the EIS responses are listed in the SI, Table S1. The charge-transfer resistance (*R*_ct_) of Ni–Fe LDH (8.4 Ω) is reduced by 50% due to graphene incorporation. A smaller *R*_ct_ indicates superior charge transport kinetics that benefits a fast OER reaction. The electrochemically active surface area (ECSA) of these two catalysts was estimated by comparing the electrochemical double-layer capacitance (*C*_dl_), as *C*_dl_ is proportional to the ECSA of electrocatalysts. The *C*_dl_ of graphene–Ni–Fe LDH is confirmed to be 1285 μF, which is more than two times higher than that of Ni–Fe LDH (536 μF).

## 3 Conclusions

In summary, the MCSS method has been reported here for rapid and gram-scale manufacturing of defect-free and processable graphene flakes from commercial graphite. The majority of LDGFs are single layer (90%) with almost no detectable defects on the surface. A thin graphene film of ∼200 nm represents an extremely low sheet resistance of *ca*. 280 Ω/sq (electrical conductivity of ∼18 000 S/m), which can be readily obtained from the LDGFs without any chemical or physical post-treatment, indicating that they are ideal materials for diverse applications in electronics, sensors, and others. The graphene–Ni–Fe LDH film represents 1 order of magnitude higher electrocatalytic activity (∼140 mA/cm^2^ at 0.7 V) for water oxidation compared to pure Ni–Fe LDH and *ca* 120 mV cathodic shift of onset potential, leading to a >90% faradic efficiency with an ideal ratio of H_2_ to O_2_ evolved due to the dramatically enhanced reaction kinetics and charge transfer by this high-quality graphene. All these advances are believed due to (i) a selective and nondestructive MI source to rapidly and extensively exfoliate P-graphite into high-quality graphene flakes in air, (ii) a self-cleaning method that effectively removes adsorbed reagents without a need of post-treatment, and (iii) a self-healing method for the defects (*e*.*g*., covalently attached surface groups) on the graphene surface, likely due to selective heating by microwave. In total, this project potentially promises a wealth of single-layer and high-quality 2D materials to be prepared by this green microwave exfoliation process and their applications in energy and environment.

## 4 Materials and Methods

Graphite was provided by Graphexel Limited and used as received. Liquid bromine (for synthesis, Fluka), chloroform (from VWR Chemicals Ltd.), *n*-decane (anhydrous assay ≥99.0%, Sigma-Aldrich), *N*,*N*-dimethylformamide (DMF) (ACS reagent, ≥99.8%, Sigma-Aldrich), and *N*-methyl-2-pyrrolidone (NMP) (99.5%, Sigma-Aldrich) were purchased from Sigma-Aldrich and used as received. High-purity distilled water was obtained from an Elga PURELAB Prima deionized water machine (15 Ω). Graphene films were prepared on PTFE membranes by in-house-developed surface tension-mediated self-assembly method (LCR Membrane Filter, PTFE, Hydrophilic, 0.5 μm, 13 mm, white, plain, Merck Millipore). FTO-coated glass (TEC 15) was provided by Pilkington NSG.

### 4.1 Preactivation of Graphite Flakes

In a typical procedure, commercial graphite flakes (2 g) were added to a sealed vial containing Br_2_/solvent solution and then left for 2 h for intercalation at room temperature. Similarly, 1, 3, and 7 day treatment terms were also used to optimize the pretreatment process. Water, chloroform, and *n*-decane were selected as solvents. P-graphite flakes were filtered through filter paper and washed with fresh chloroform to remove the remaining Br_2_ molecules and then was transferred into a clean vial and dried at room temperature in a fume cupboard. Dried samples were labeled as the P-graphite and stored at room temperature in a sealed glass vessel.

### 4.2 Special Mode Microwave-Controlled Irreversible Solid-State Exfoliation of the P-Graphite

In a typical procedure, 20–100 mg of the P-graphite was transferred into a sealed glass tube and irradiated by a microwave irradiation for about 10 s, using a CEM microwave fitted with an Infrared (IR) temperature sensor. The temperature of the solid reaction material was recorded as 360 ± 20 °C. The reaction medium was cooled to 50 °C, and the glass tube was opened in a fume cupboard. Highly exfoliated solid graphitic material was obtained and labeled as EG. The EGs were further dispersed in organic solvents (DMF or NMP) or aqueous solution with a surfactant (sodium dodecylbenzenesulfonate (SDBS), cetyltrimethyl-ammonium bromide (CTAB), or Triton X-100 (TX-100)) to produce a highly processable LDGFs, containing ∼90% SLGFs.

### 4.3 Synthesis of Ni–Fe Layered Double Hydroxide (LDH) Electrodes

The Ni–Fe LDH was synthesized by a hydrothermal method according to a previous report.^45,47^ Similarly, 0.3 g [Ni(NO_3_)_2_], 0.4 g [Fe(NO_3_)_2_], and 0.3 g urea were mixed in 80 mL of deionized water. Following dissolution, 20 mL of solution was transferred into a 50 mL Teflon autoclave with a piece of Ni foam (washed in 5 M HCl prior to use) rested against the wall. The growth of the Ni–Fe LDH catalyst was carried out at 120 °C in an electric oven for 12 h. After cooling to room temperature, the samples were removed, washed with deionized water, and dried under ambient conditions. Catalyst loading mass was measured on average as 0.2 ± 0.02 mg/cm^2^. To synthesize graphene–Ni–Fe LDH electrodes, the EG (3 mg) in DMF (10 mL) was sonicated for 1 h. The supernatant was added to a 1:1 water isopropanol mix (3 mL) containing 50 μL of 5% Nafion perfluorinated resin. The mixture was sonicated for a further 30 min, then 1 mL of solution was drop-cast on the HCl-rinsed Ni foam substrates, and then dried under a vacuum oven at 120 °C. These graphene–Ni foam substrates were then used for hydrothermal deposition of Ni–Fe LDH.

### 4.4 Electrocatalytic Activity of Graphene Electrodes

Oxygen evolution reaction (OER) measurements were conducted using a gas-tight single-compartment three-electrode electrochemical cell (Adams-Schittenden Co) linked to a potentiostat (Ivium Technology). The samples, deposited on Ni foam, were attached to a stainless steel alligator clip (RS components) with the nonexposed area coated with an ultraresistant ATACS epoxy resin and used as the working electrodes (the exposed area = 0.5 cm × 0.5 cm). Pt mesh and Ag/AgCl (3 M KCl) were used as counter electrode and reference electrode, respectively. The scan speed was 20 mV/s, and the electrolyte was 1 M KOH (pH 14) (reagent grade, 90%, Sigma-Aldrich). For H_2_ and O_2_ evolution, the cell was sealed and purged by argon gas (99.999%, BOC) for 1 h prior to testing. Evolved gaseous H_2_ and O_2_ amounts were detected by an off-line gas chromatograph (Varian GC-430, argon carrier, 99.999%) equipped with a TCD detector using a gas-tight syringe, *e*.*g*., manually taking 0.5 mL of gas samples from the reactor headspace using the 1 mL syringe (SGE, Australia).

### 4.5 Material Characterization

Raman spectra were obtained from a Renishaw InVia Raman Microscope, using a 514.5 nm excitation laser and wavenumber ranging between 100 and 3000 cm^–1^. Commercial graphite, the P-graphite, and EG were tested in powder form. A stable DMF dispersion of graphene flakes (LDGF) was deposited on the substrate surface and tested in powder form. Raman analysis was performed on a piece of clean Si-wafer substrate to prevent the interference of the substrate to the spectra and to observe any peak shift relative to the Si reference material at 520 cm^–1^. UV/vis spectra were obtained from stable dispersions of the produced graphene in organic or aqueous solutions using a Shimadzu UV-2550 UV/vis spectrophotometer. High-resolution XPS was performed by a Thermo Scientific K-Alpha photoelectron spectrometer with monochromatic Al Kα radiation; peak positions were referenced to the C 1s line at 284.5 eV, and CasaXPS software was used for data processing. Samples for AFM analysis were produced by drop deposition (4 μL) onto the Si-wafer of the corresponding solution of graphene flakes (*ca*. 0.005 mg/mL) in DMF. Samples were dried in air before imaging in tapping mode using a Digital Instruments Multimode AFM instrument with a Nanoscope IV controller. X-ray diffraction (XRD) was performed using a Stoe STADI-P diffractometer using Cu Kα_1_ (using 40 kV and 30 mA) radiation (λ = 1.54 Å). Diffraction patterns were collected from 5 to 90°, and a step size of 0.09° s^–1^ was used. Scanning electron microscopy (SEM) of graphene films was carried out using a JEOL-6700M field-emission scanning electron microscope, operated in gentle beam mode (6 mm stage height, 0° stage tilt, 2 kV acceleration voltage, 10 mA current) to minimize surface charging effects. Transmission electron microscopy (TEM) and high-resolution transmission electron microscopy (HRTEM) were performed using Jeol JEM-1010 and JEOL-2010F coupled with an EDS detector (Oxford Instruments) instruments, respectively. For hexagonal lattice analysis of graphene flakes dispersed in DMF and deposited on TEM grid, high-resolution transmission electron microscopy was performed on an aberration-corrected FEI Titan operated at 80 kV. To achieve low-noise images, each area was captured approximately 15 times with 1 s exposures and subsequently aligned and then summed. An average background subtraction filter was then applied to remove the smoothly varying background due to uneven illumination across the full image. TEM samples were prepared from stable dispersions of graphene flakes prepared in DMF by dropping on carbon-coated copper grids, which were dried in air before imaging. A four-probe electrical conductivity of graphene films on PTFE membrane was measured using a Keithley 2450 SourceMeter at six different locations on the sample. All graphs and statistical analysis were obtained using OriginLab software (Origin 9.1).

## Supporting Information Available

Effect of solvent (water, chloroform, and *n*-decane) and time (2 h, 1, 3, and 7 days) on the activation of graphite *via* Br_2_; characterization of (non)-aqueous dispersions of the graphite and EG; Raman spectra of the P-graphite and EG; both characteristic Br_2_ harmonic Raman signals and G-band splitting after pretreatment indicate the success of the intercalation process; HRTEM image of LDGF, which was performed on an aberration-corrected FEI Titan HRTEM operating at 80 kV; and TEM images of different LDGFs (PDF)
