# Supplementary material for: Defect-Free Single-Layer Graphene by 10 s Microwave Solid Exfoliation and Its Application for Catalytic Water Splitting
Source: ACS Appl Mater Interfaces. 2021 Jun 10;13(24):28600–9. doi: 10.1021/acsami.1c03906 (PMC8289231; doi:10.1021/acsami.1c03906)
Supplement: Supplementary file 1 — am1c03906_si_001.pdf [file am1c03906_si_001.pdf]

Supporting Information

Defect-Free Single Layer Graphene by 10 s Microwave Solid Exfoliation and Its Application for Catalytic Water Splitting

Mustafa K. Bayazit[1±] , Lunqiao Xiong[1], Chaoran Jiang[1], Savio J. A. Moniz[1], Edward White[2], Milo S.P. Shaffer[2] and Junwang Tang[1]*

[1] Department of Chemical Engineering, University College London, Torrington Place, London WC1E 7JE, UK.

[2] Department of Chemistry, Imperial College London, London SW7 2AZ, UK

[±] Sabanci University Nanotechnology Research and Application Center, Tuzla, Istanbul 34956, Turkey. (Present Address)

Corresponding Author's E-mail: junwang.tang@ucl.ac.uk

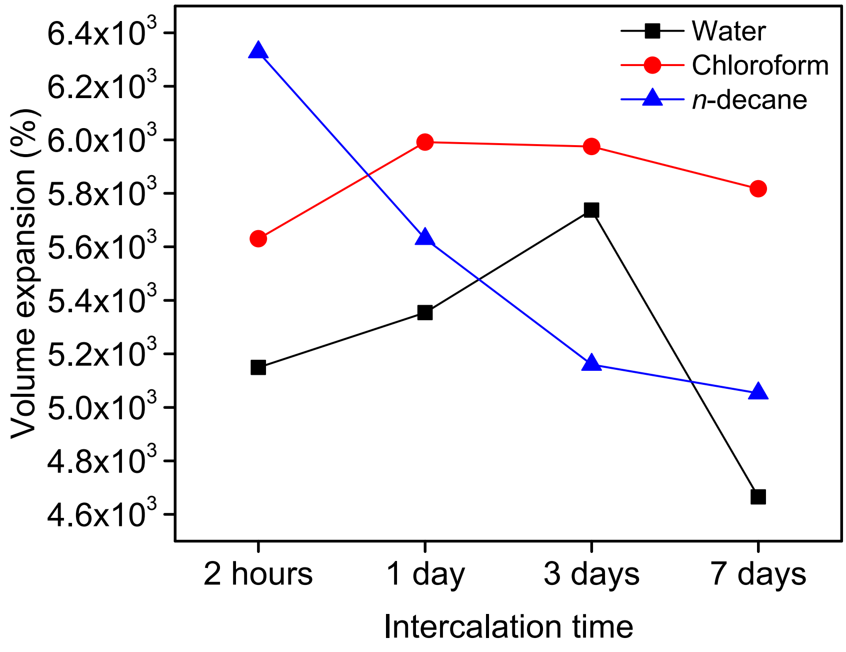

Fig. S1. The effect of solvent (water, chloroform and *n*-decane) and time (2h, 1 day, 3 days and 7 days) on the activation of graphite via Br_2. The solid volume expansion after special mode microwave irradiation (SMI) exfoliation is used as a key factor to illustrate this effect. Pre-treatment of graphite by Br_2 in chloroform looks to be a stable process compared to water and *n*-decane, and does not change significantly by time.

The influence of solvent and pre-treatment time on the degree of final exfoliation by SMI were investigated using a range of solvents such as deionized water (H_2O), chloroform ($CHCl_3$) and *n*-decane ($C_{10}H_{22}$), as well as different time intervals (2 h, 1 day, 3 days, 7 days). Experimentally acquired solid volumes of the obtained EGs were used to express the degree of exfoliation. The highest volume expansion (approximately 64x larger than the estimated volume of the as-received graphite using the bulk graphite density of 2.1 g/cm^3) was obtained from the sample pre-treated in *n*-decane for 2 hours, compared to the P-graphites prepared in H_2O (52x) and $CHCl_3$ (56x). The P-graphites prepared in $CHCl_3$ was used throughout this study as it was relatively stable.

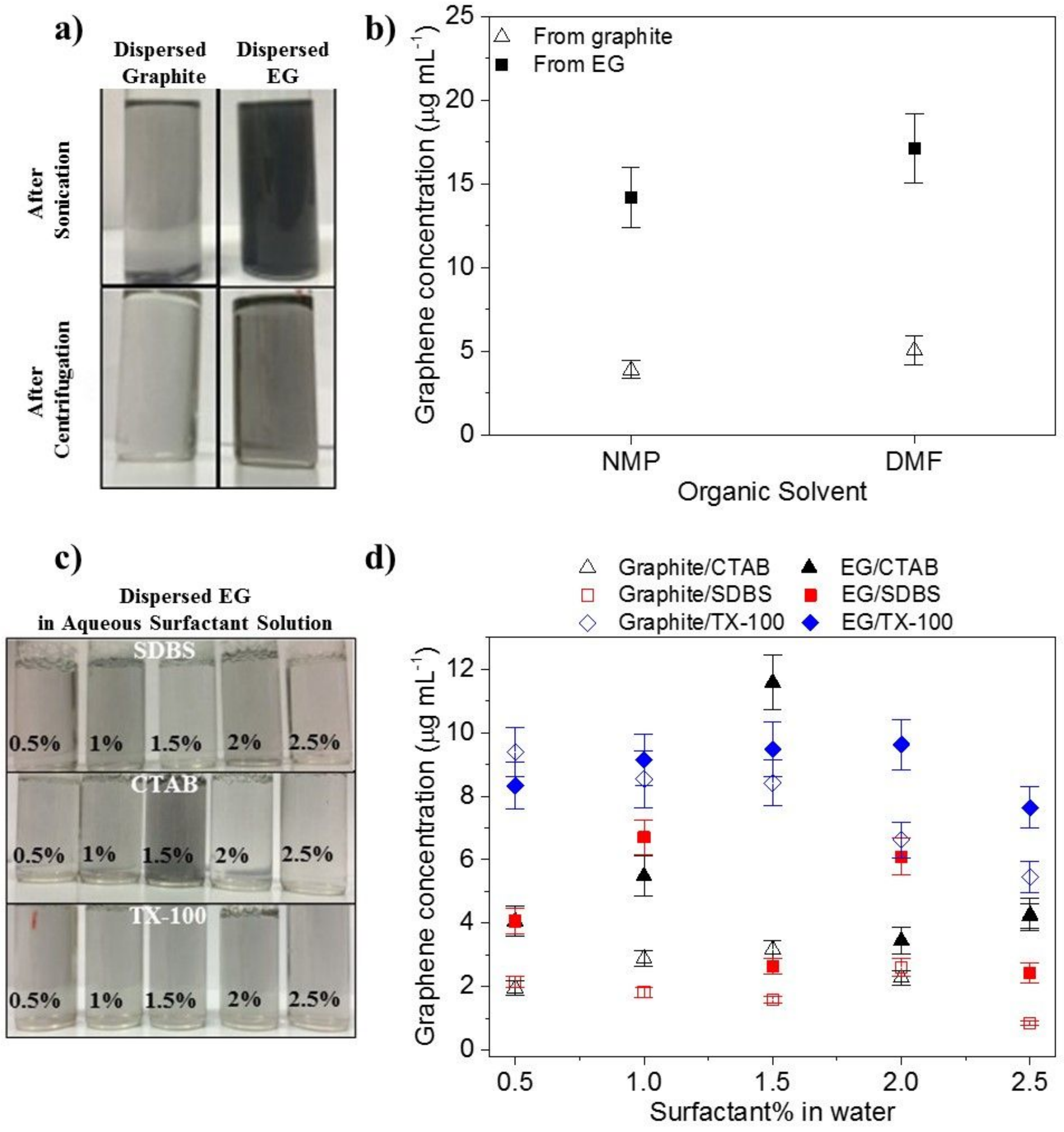

Fig. S2 Characterization of (non)-aqueous dispersions of the graphite and EG. a) Photographs of the as-received graphite and EG dispersions in DMF. The highest processable concentration (approximately 4-fold higher than the as-received graphite) was obtained in DMF using the EG. A similar dispersion color was observed in NMP (not shown). **b)** Graphene concentration in organic solvents (NMP and DMF). Concentrations were estimated from the absorbance value observed at 660 nm using the Beer-Lambert equation and the reported molar absorptivity coefficient of 2,460 L g^{-1} m^{-1} [1]. **c)** Photographs of the graphene flakes dispersions with various weight percentages (0.5, 1, 1.5, 2.0 and 2.5 wt%) of aqueous SDBS, CTAB and

TX-100 dispersions. The highest processable concentration is obtained in 1.5 wt.% aqueous CTAB solution. **d)** Graphene concentration in aqueous SDBS, CTAB and TX-100 solutions with various weight percentages (0.5, 1, 1.5, 2.0 and 2.5 wt.%). The *y-axis* error bars refer to the standard deviation obtained from three measurements.

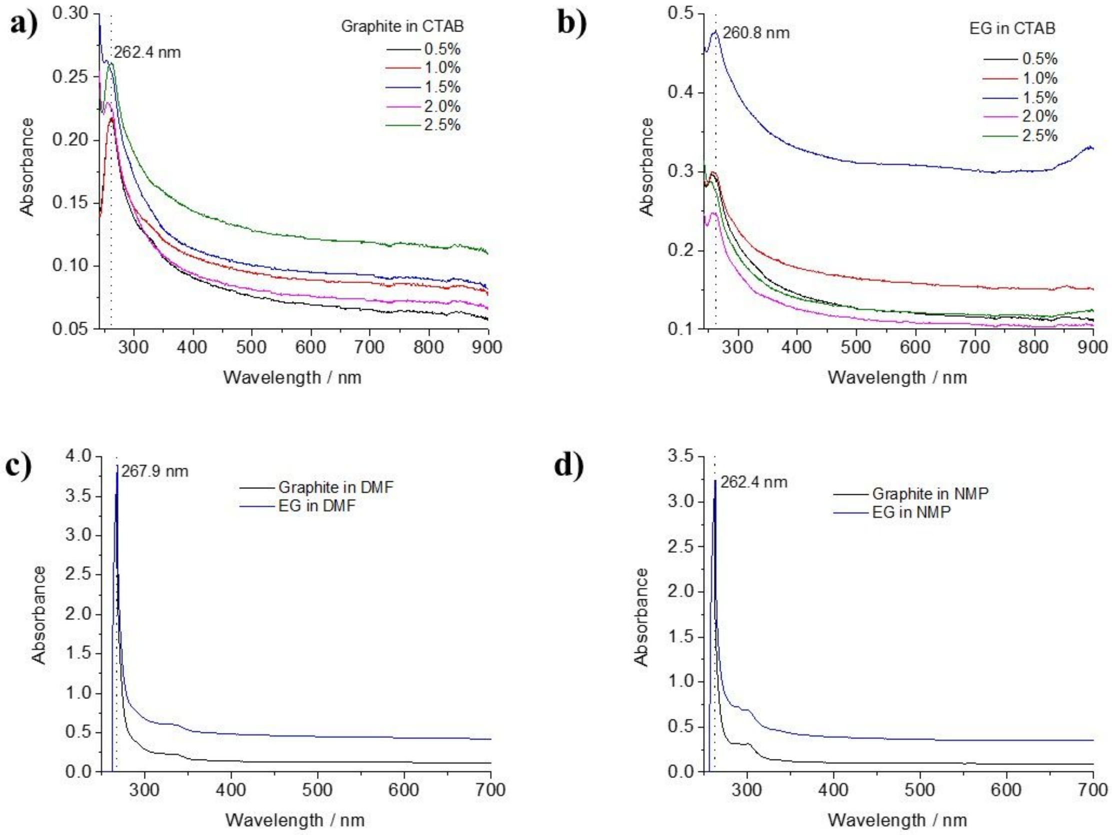

Fig. S3 UV-vis spectra of the as-received graphite and EG in various weight percentages (0.5, 1, 1.5, 2.0 and 2.5 wt%) of CTAB aqueous solution (**a** and **b**), **c)** DMF and **d)** NMP.

The EGs were dispersed in two different organic solvents (NMP and DMF) and three different surfactant-containing (SDBS, CTAB and Triton X-100) aqueous solutions. As a control experiment, solutions of the as-received graphite were also prepared in the same solvent by subjecting them to the similar experimental conditions. In a typical experiment, the graphitic material was dispersed at a concentration of 1 mg/mL in an ultra-sonication bath for 60 minutes. The resultant dispersion was then centrifuged for 90 minutes at 500 rpm. In all cases, a grey-colored dispersion, slightly varying in appearance from light to dark, was obtained, and it was further centrifuged to extract highly dispersible graphene flakes (**ESI Fig. S2a** and **c**). The

concentration of the prepared graphitic material dispersions was calculated from their UV–vis absorption spectra at 660 nm following the Beer-Lambert equation and using the reported molar absorptivity coefficient of 2,460 L g^{-1} m^{-1} (see **ESI Fig. S3** for UV-vis spectra of the as-received graphite and EG in aqueous surfactant solutions and organic solvents) [1]. The concentrations of graphene solutions are given in **Fig. S2 b** and **d**. Mild sonication of EG in pure organic solvents, DMF and NMP, yielded the highest graphitic material concentration, compared to the as-received graphite under the same conditions. The concentration of the homogeneous grey solutions of the graphene flakes in DMF and NMP were similar, likely due to their comparable surface tension values which are 37.1 and 40.1 mJ/m^2, respectively [1]. The highest calculated concentration value for the graphene flakes was obtained as 17.1 (±2.1) µg mL^{-1} in DMF (~14 µg mL^{-1} in NMP), approximately four times higher than the obtained graphitic material concentration when the as-received graphite (5.1±0.86 µg mL^{-1}) was used. The observed increase in concentration could be related to both the weakening of the strong π-π interactions between graphitic layers and the solid-state exfoliation of significant amounts of graphitic layers after the microwave-controlled irreversible fast exfoliation of the PG.

The nature of surfactant, its concentration and type of interaction are known to be crucial when defining the phase behavior of carbon nanomaterials, especially nanotubes and graphene. In order to explore the effectiveness of the aqueous surfactant systems to produce graphene-like materials from the as-received graphite and graphene flakes under the identical experimental conditions, the solubility of graphitic materials in three different surfactants (anionic (SDBS), a cationic (CTAB) and a non-ionic (Triton X-100)) was studied using 0.5, 1, 1.5, 2 and 2.5 wt.% surfactant. Stable graphitic solutions are presented in **Fig. S2c**. In agreement with the solubility data obtained in this work in the organic solvents, the graphene flakes showed the highest solubility in the surfactant-containing aqueous solutions after dispersion compared to the as-received graphite. The highest solubility of the graphene flakes was recorded in 1.5 wt%

in the cationic surfactant (CTAB) solution, suggesting a strong interaction between the hydrophobic cation and the graphene flakes. In contrast, the low solubility obtained in the anionic surfactant (SDBS) indicated a weak interaction between SDBS and the graphene flake surface. Very recently, it has been shown that graphene flakes were negatively charged in water, confirmed by the electrophoretic mobility and zeta potential measurements [2]. It is believed that the solid-state exfoliation of the PG produces a significant amount of graphene flakes, whose surfaces are also negatively charged in deionized water, facilitating the strongest interaction between CTAB cations and graphene but weakest between SDBS and graphene.

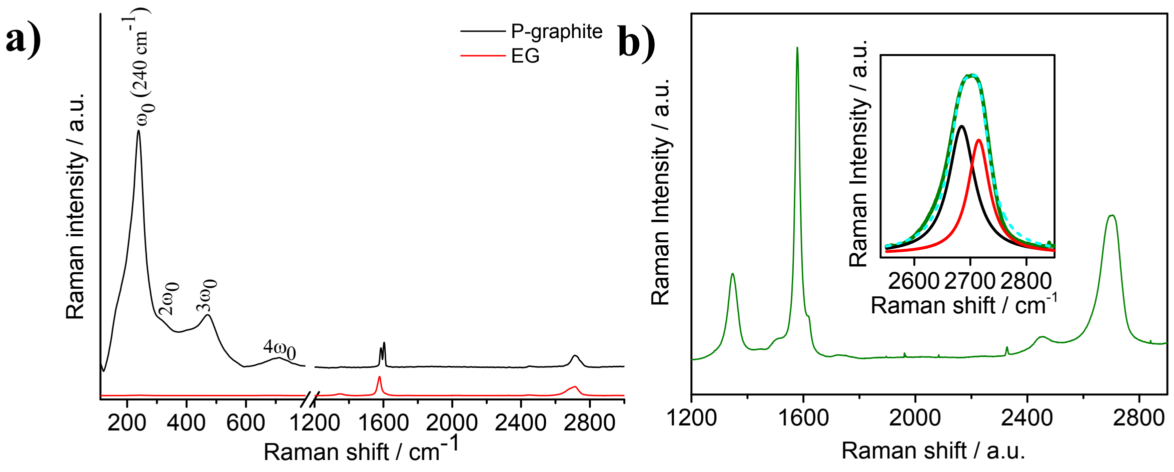

Fig. S4 a) Raman spectra of the P-graphite and EG. Both characteristic Br_2 harmonic Raman signals and *G-band* splitting after pre-treatment indicate the success of the intercalation process. Raman spectrum of the EG shows no Br_2 peak and G-splitting, indicative of the removal of intercalated Br_2. **b)** Raman spectrum of the LDGFs. LDGFs exhibit an increased I_D/I_G ratio of 0.35, comparable with the I_D/I_G ratio of edge functionalized small graphene flakes, probably due to the sonication process [3]. Inset: The *2D-band* Raman spectrum of the LDGFs (from 62 different graphene flakes) shows the characteristic Raman peak shape of bilayer graphene and can be fitted by two Lorentzian peaks [4]. These bilayer flakes are likely to be formed by the re-

stacking of SLGFs during the sample preparation for Raman analysis. Dash line represents fit line.

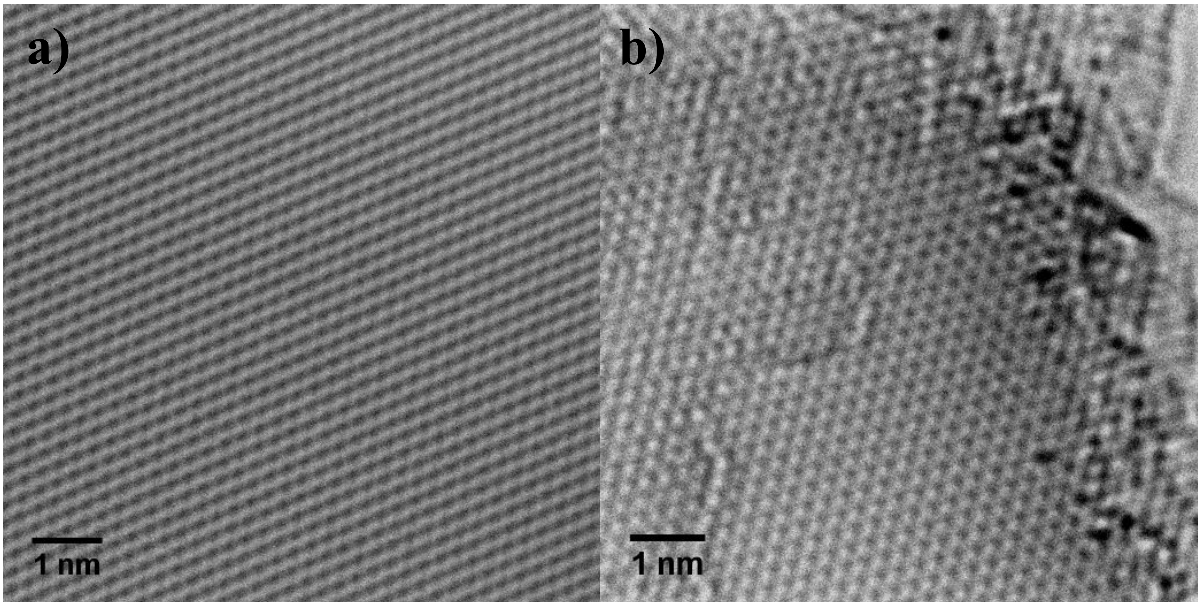

Fig. S5 HRTEM image of LDGF which was performed on an aberration-corrected FEI Titan HRTEM operating at 80 kV. Each image was obtained from different graphene flakes. The image (**a**) again shows a defect-free hexagonal lattice, providing further evidence that the SMI is a non-destructive solid-state exfoliation process. The image **b** also shows a defect-free hexagonal lattice of probably re-stacked graphene flakes.

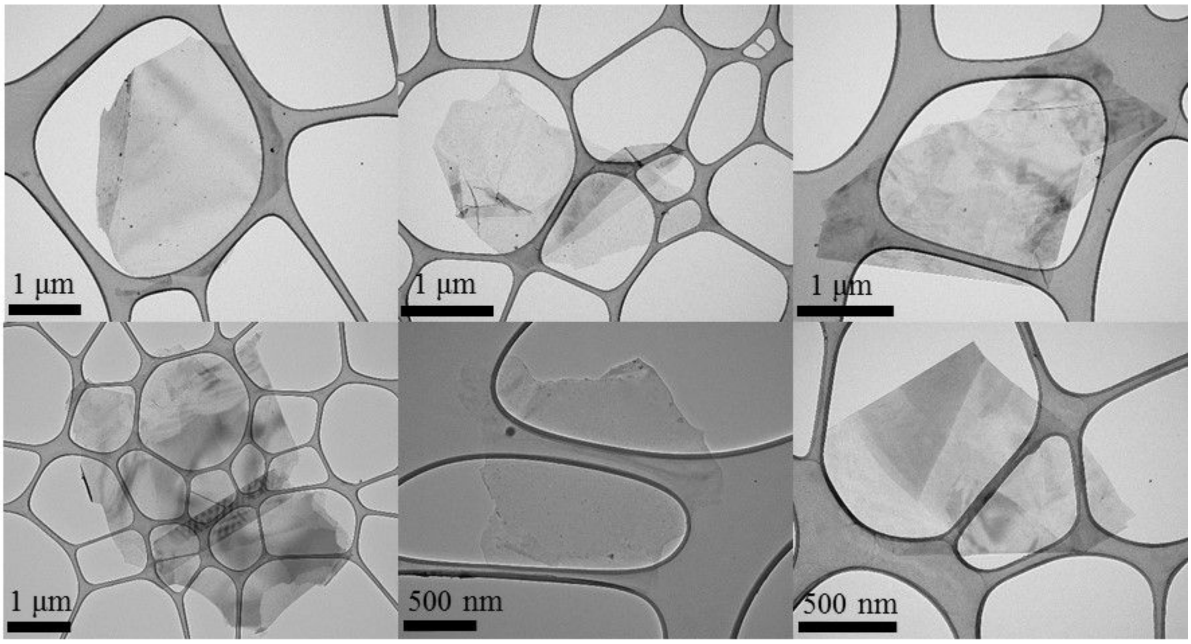

Fig. S6 TEM images of different LDGFs. The images show graphene flakes with the lateral dimensions of 2-5 μm. The transparent graphene flakes together with the random stacking suggest that produced LDGFs are highly individualized in solvent.

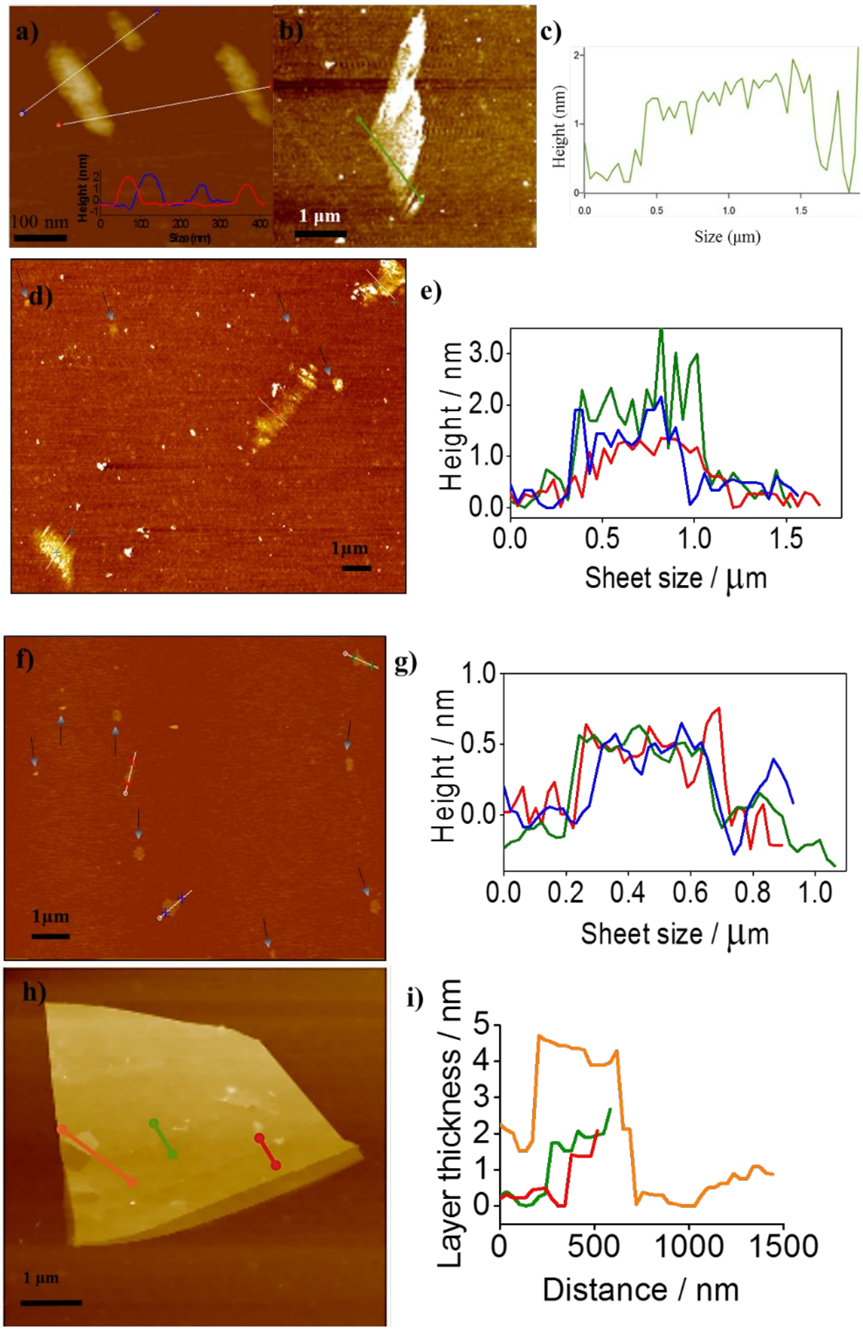

Fig. S7 a) AFM image shows single- and bilayer graphene flakes. Inset shows the height profile of the selected graphene flakes. Thicknesses of graphene flakes are comparable with the SLGF thicknesses reported. **b)** AFM image of a probably bilayer graphene with lateral dimension of ~4.0µm. **c)** Height analysis of the bilayer graphene in Fig. S8b. We believe that measured height slightly higher than 1nm is consistent with bilayer graphene height although it is

attributed to SLGFs in various studies [1, 5]. **d)** Typical AFM height images of graphene flakes with large lateral dimensions of 2-5 μm. **e)** The measured height of samples in **d**, by step analysis, marked by straight lines (red, blue and green) with cross-arrow heads [1, 6]. The flake (green line) slightly thicker than 1.0 nm shows bilayer graphene while the flakes (red and blue lines) ≤1 nm show SLGFs. **f)** Typical AFM height images of graphene flakes with lateral dimensions smaller than 1 μm. **g)** Layer thickness distribution of single-layer graphene sheets with lateral sizes smaller than 1 μm. **h)** Typical AFM height images of graphene flakes with large lateral dimensions of 2-5 μm. **i)** Layer thickness analysis, marked by straight lines (red, orange and green), of stacked graphene sheets with larger lateral sizes.

Upon further layer thickness analysis performed by counting 27 individual graphene sheets revealed that average thickness of ~90% of the LDGFs were under ~1.0 nm, but their sizes were less than ~1.0 μm **w**hereas graphene flakes with lateral dimensions of ~1.0 to 5.0 μm were slightly thicker than ~1.0 nm, attributed to bilayer graphene.

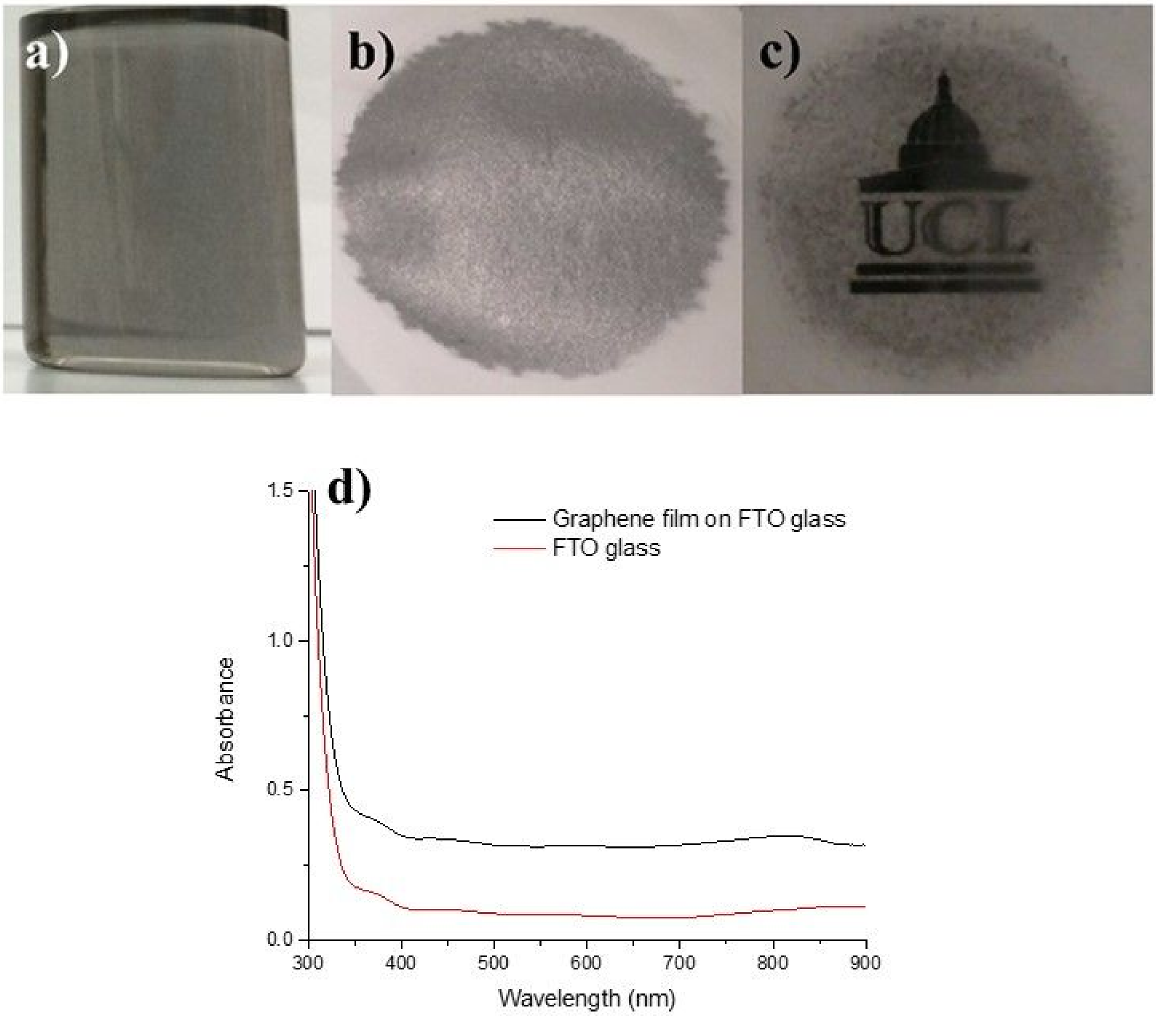

Figure S8 a) LDGFs in DMF (10 μg/mL), **b)** Graphene film on PTFE membrane prepared via vacuum-filtration of the LDGFs (6 mL) in DMF. (Diameter ≈ 1.3 cm) and **c)** Photograph of the transparent graphene film transferred onto a polystyrene substrate. The UCL logo under the polymer substrate coated with a *ca.* 200 nm graphene thin film is clearly visible, revealing the transferred film is transparent. **d)** UV-vis spectra of the transferred graphene film on the FTO glass and the FTO glass as a reference.

Table S1. The impedance parameters derived by fitting the EIS responses on Ni-Fe-LDH and graphene-Ni-Fe-LDH in 1 M KOH at an overpotential of 230 mV.

Ni-Fe-LDH		graphene-Ni-Fe-LDH	
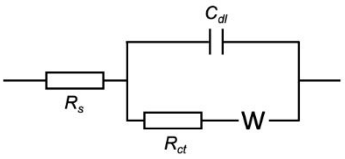		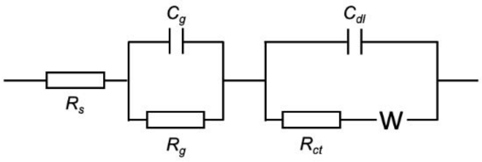	
R_s (Ohm)	1.6	R_s (Ohm)	1.7
R_{ct} (Ohm)	8.4	R_{ct} (Ohm)	4.4
C_{dl} (μF)	536	C_{dl} (μF)	1285
		R_g (Ohm)	10.3
		C_g (μF)	554

Electrochemical impedance spectroscopy (EIS) measurements were performed over a frequency range from 1 Hz to 100 kHz at an overpotential of 230 mV.

(1) Hernandez, Y.; Nicolosi, V.; Lotya, M.; Blighe, F. M.; Sun, Z.; De, S.; McGovern, I. T.; Holland, B.; Byrne, M.; Gun'ko, Y. K.; Boland, J. J.; Niraj, P.; Duesberg, G.; Krishnamurthy, S.; Goodhue, R.; Hutchison, J.; Scardaci, V.; Ferrari, A. C.; Coleman, J. N. High-Yield Production of Graphene by Liquid-Phase Exfoliation of Graphite Nat. Nanotechnol. 2008, 3, 563-568.
(2) Liu, M.; Zhang, R.; Chen, W. Graphene-Supported Nanoelectrocatalysts for Fuel Cells: Synthesis, Properties, and Applications Chem. Rev. 2014, 114, 5117-5160.
(3) Casiraghi, C.; Hartschuh, A.; Qian, H.; Piscanec, S.; Georgi, C.; Fasoli, A.; Novoselov, K. S.; Basko, D. M.; Ferrari, A. C. Raman Spectroscopy of Graphene Edges Nano Lett. 2009, 9, 1433-1441.
(4) Malard, L. M.; Pimenta, M. A.; Dresselhaus, G.; Dresselhaus, M. S. Raman Spectroscopy in Graphene Phys. Rep. 2009, 473, 51-87.
(5) Khan, U.; O'Neill, A.; Lotya, M.; De, S.; Coleman, J. N. High-Concentration Solvent Exfoliation of Graphene Small 2010, 6, 864-871.
(6) Paton, K. R.; Varrla, E.; Backes, C.; Smith, R. J.; Khan, U.; O'Neill, A.; Boland, C.; Lotya, M.; Istrate, O. M.; King, P.; Higgins, T.; Barwich, S.; May, P.; Puczkarski, P.; Ahmed, I.; Moebius, M.; Pettersson, H.; Long, E.; Coelho, J.; O'Brien, S. E.; McGuire, E. K.; Sanchez, B. M.; Duesberg, G. S.; McEvoy, N.; Pennycook, T. J.; Downing, C.; Crossley, A.; Nicolosi, V.; Coleman, J. N. Scalable Production of Large Quantities of Defect-Free Few-Layer Graphene by Shear Exfoliation in Liquids Nat. Mater. 2014, 13, 624-630.
